# A 15-Year Ecological Comparison for the Hiring Dynamics of Minnesota Pharmacies between 2006 and 2020

**DOI:** 10.3390/pharmacy9020100

**Published:** 2021-05-06

**Authors:** Jon C. Schommer, Anthony W. Olson, SuHak Lee, Caroline A. Gaither, Stephen W. Schondelmeyer

**Affiliations:** 1 College of Pharmacy, University of Minnesota, Minneapolis, MN 55455, USA; leex6829@umn.edu (S.L.); cgaither@umn.edu (C.A.G.); schon001@umn.edu (S.W.S.); 2 Research Division, Essentia Institute of Rural Health, Duluth, MN 55805, USA; anthony.olson@essentiahealth.org

**Keywords:** pharmacist, technician, pharmacy, workforce, hiring, salary, employment

## Abstract

Labor market forces in pharmacy are affected by frictional unemployment (job turnover), structural employment forces that require new skill sets for employees, and hiring practices that integrate technology or less costly labor such as pharmacy technicians. The objectives of this study were to describe hiring trends for both the pharmacist and technician workforces in licensed pharmacies on a biennial basis from 2006 through 2020 using data collected in Minnesota. Ecological comparisons were made between the survey years using descriptive statistics. For open-ended questions added to the 2020 survey, content analysis was applied. Demand for technicians increased which might be due to the expansion of their roles into activities that had been reserved for the pharmacist. Pharmacies reportedly would like to hire pharmacists to meet the demand for new services that pharmacists can provide. However, respondents articulated that this is not feasible under current economic pressures. This represents a lost opportunity for transformation in pharmacy that would establish pharmacists’ roles in the rapidly transforming health care value chain. We conclude that hiring dynamics in pharmacies are being driven more by economic and organizational shifts than meeting the demand for services that pharmacists can provide.

## 1. Introduction

**Figure 1:**
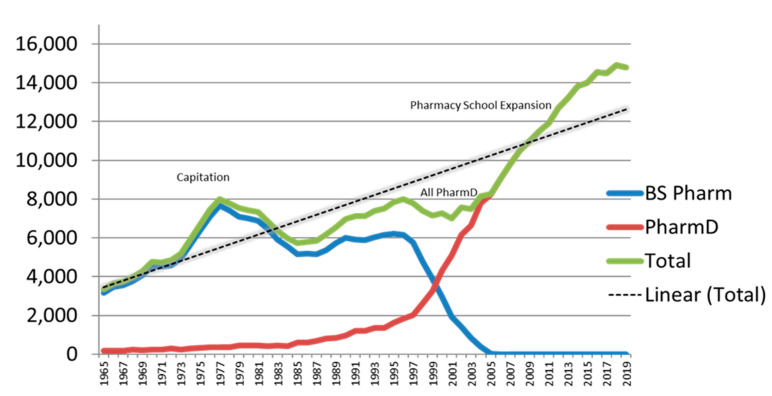
Pharmacy Degrees Conferred in the United States as First Professional Degree (1965–2019). Source: 2019 Profile of Pharmacy Students—AACP https://www.aacp.org/sites/default/files/2020-05/fall-2019-pps-enrollments.pdf, accessed 19 January 2021. Capitation: pharmacy schools and colleges received “capitation grants” on the basis of size of enrollment and numbers of graduates through the Comprehensive Health Manpower Training Act of 1971 [6] and the Health Professions Educational Assistance Act of 1976 [7] which expired in fiscal year 1980. All PharmD: conversion to the PharmD as the only first professional degree introduced gaps in graduation classes for some schools and colleges of pharmacy and decreased the total number of graduates in the United States during that time period. From 2004 onward, “PharmD” and “Total” lines in the graph are the same. Pharmacy School Expansion: the number of pharmacy schools in the United States expanded from 82 in 2000 to 143 in 2019 (www.aacp.org), accessed 19 January 2021.

From 2000 to 2019, National Pharmacist Workforce Surveys monitored the size, demography, and activities of the pharmacist workforce in the United States [1,2,3,4,5]. Figure 1 represents the supply side of this appraisal by summarizing the number of pharmacy degrees conferred in the United States as the first professional degree from 1965 through 2019 and is useful for interpreting the findings from the national surveys. The “Capitation Years” in the 1970s resulted in growth in the number of pharmacy degrees conferred, during which time pharmacy schools and colleges received “capitation grants” on the basis of enrollment size and numbers of graduates through the Comprehensive Manpower Training Act of 1971 [6] and the Health Professions Education Assistance Act of 1976 [7]. These grants expired in fiscal year 1980, corresponding with a reduction in numbers of graduates per year, which were followed by another reduction in the late 1990s and early 2000s when degrees conferred transitioned to the “All PharmD” (see Figure 1). Between 2006 and 2010, “Pharmacy School Expansion” [8] took place to address pharmacist shortages produced from a combination of increased retirements by pharmacists trained during the “Capitation Years” and a shortage of substitutes produced during what scholars call “Retrenchment Years” (1980 through 2005) [1,2,3,4,5,8,9]. Such periods of expansion and retrenchment were commonplace during the 20th century from influences such as the Great Depression, World War II, the GI Bill, changes in accreditation requirements, and education assistance acts [8]. However, the steepest slope in the accumulation of new pharmacy schools (since 1900) was between 2006 and 2010 [8], with continued expansion of both schools and graduates through 2015 (Figure 1).

The most recent expansion reduced the shortage of pharmacists experienced in the early 2000s [1,2,3,4,5,9,10]. For example, the Aggregate Demand Index (ADI) for pharmacists from 2002 to 2008 had been in the 4 to 5 range (i.e., high demand for pharmacists nationally) [9]. From 2009 to 2015, the ADI dropped to the 3 to 4 range (i.e., moderate demand), and then to the level of 3 (i.e., demand in balance with supply) from 2016 to 2018 [10].

**Figure 2:**
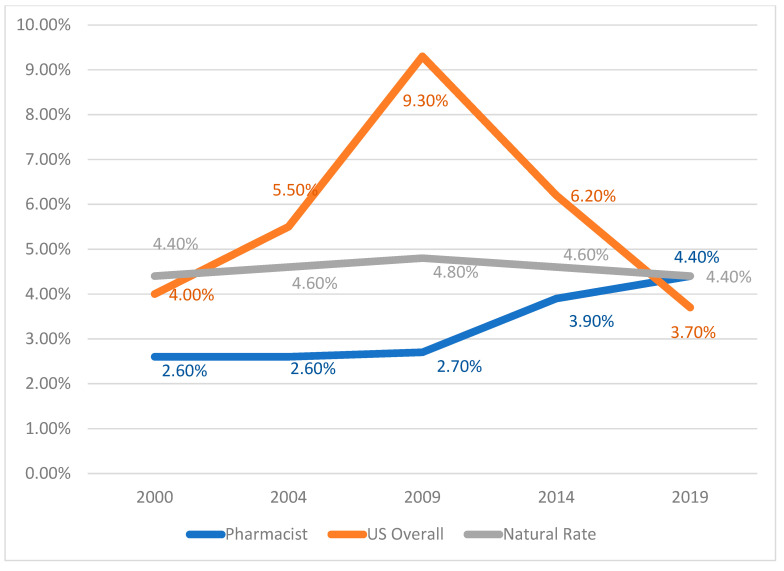
United States Unemployment (Pharmacist, Overall, and Natural) from 2000 to 2019. Pharmacist unemployment estimated from National Pharmacist Workforce Surveys [1,2,3,4,5]. Overall unemployment estimated from U.S. Bureau of Labor Statistics, Division of Labor Force Statistics, Washington, DC 20212-0001, www.bls.gov/CPS accessed 17 February 2021 [11]. Natural unemployment estimated from U.S. Congressional Budget Office, Natural Rate of Unemployment (Long-Term) (NROU), retrieved from FRED, Federal Reserve Bank of St. Louis; https://fred.stlouisfed.org/series/NROU, accessed 17 February 2021 [12,13,14].

Figure 2 shows that pharmacist unemployment in the United States was less than 3% for the years 2000, 2004, and 2009 [1,2,3]. These national unemployment levels for pharmacists were less than both the national unemployment rate overall [11] and “natural unemployment” estimates from the United States Congressional Budget Office [12], which is the lowest level that a healthy labor market can sustain without creating inflation [12,13,14,15]. By 2014, pharmacist unemployment was 3.9% [4], which was closer to, but still below, the natural unemployment rate estimate [12,13,14,15]. Pharmacist unemployment in 2019 reached 4.4% [5], which was equal to the natural unemployment rate estimate of 4.4%.

The ideal rate of unemployment (natural unemployment) means the labor market and economy are strong and jobs are available [12,13,14,15]. When a labor market is healthy, natural unemployment results from normal turnover (frictional unemployment). People who are frictionally unemployed might include new graduates seeking their first job, employees who decide to leave a position to move to a new town before finding a new job, or others who leave the workforce for personal reasons. Natural unemployment is also associated with structural unemployment in which people have skill sets that become outdated, or their jobs are being replaced by technology or less costly labor. Structural unemployment remains until workers receive new training. When “overall unemployment” is below “natural unemployment”, inflationary forces create an overheated labor market linked to precipitous increases in wage rates and student debt loads, as well as a decreased incentive for workers to keep their job skills from becoming outdated.

Pharmacy scholars correctly predicted that retrenchment in the number of pharmacy graduates was needed before the end of the 2010s [1,2,3,4,5,8,9]. Further, they described the need for continual transformation in pharmacy practice and education that would update job skills for pharmacist contributions to health care and patient wellbeing [8,9,16,17,18,19]. There are signals that the number of pharmacy degrees conferred per year is decreasing (2019 Profile of Pharmacy Students—AACP). Furthermore, the majority of pharmacists in the United States (52% in 2019) now contribute a significant portion of their time to providing patient care services that are distinct from the medication dispensing process (up from 40% in 2014) [20]. However, Lebovitz and Rudolph adroitly pointed out that “large-scale practice transformation will not happen overnight; consequently, schools and colleges of pharmacy must immediately change their perspective … to producing graduates who are prepared with expertise and professional skills to excel in many types of well-paying positions” [17]. Van Antwerp [21] proposed that the health care value chain, and pharmacy’s role in it, will experience a rapid disruption into areas such as: gene therapy, microbiomics, digital therapeutics, central-fill delivery hubs, ingestible robotics, home health diagnostics, automated artificial intelligence algorithms, smart home technology, and social determinants of health. Thus, transformations in pharmacy practice and education will likely be both dynamic and disruptive. 

To better understand labor market forces in pharmacy, we propose that it would be helpful to describe frictional unemployment (job turnover) and structural employment forces that require new skill sets for employees and hiring practices that are affected by technology or less costly labor such as pharmacy technicians [16,17,18]. This will help inform decision making by policy-makers, employers, and educators about skills development, novel career pathway planning, innovative training options, scope of practice updates, alignment of payment policies, tiered degree systems, and post-graduate training and credentialing [9,16,17,18,19].

In order help fill this gap in understanding, the objectives of this study were to describe hiring trends for both the pharmacist and technician workforces in licensed pharmacies on a biennial basis from 2006 through 2020 using data collected in Minnesota [22,23,24,25,26,27,28,29,30,31]. Minnesota was selected based on the availability of longitudinal data over the span of 15 years. For 2006 through 2020, hiring trends were described in terms of (1) degree of difficulty to hire pharmacists and technicians, (2) the wage rates the pharmacy market is willing to offer pharmacists and pharmacy technicians, (3) ideal and realistic demand for pharmacists and technicians, and (4) job turnover for pharmacists and technicians. 

In light of the dynamic pharmacy labor market and the COVID-19 pandemic, five open-ended questions were added to the 2020 survey. They were as follows:

What are the most impactful services available at your pharmacy?

What are the ideal characteristics of your next pharmacist hire?

What things do your pharmacy staff members need the most right now?

What things does your community/the people you serve need the most right now?

How has the COVID-19 pandemic affected your pharmacy?

Findings from the answers to these questions can provide insights for how pharmacies are conducting their hiring practices for adding new skill sets, within the rising prominence of the roles filled by technology and technicians. 

## 2. Methods

### 2.1. Description of Study Area: Minnesota Population

During the time of this study (2006 to 2020) [32], Minnesota’s population grew from 5.2 million in 2006 to an estimated 5.6 million in 2019, of whom 85% in 2006 and 80% in 2019 were White, non-Hispanic. In 2006, 12% of the population were aged 65 or older and this grew to 16% by 2019 (most recent data). Median household income increased from USD 66,896 in 2006 to USD 74,593 in 2019. In 2006, 61% resided in owner-occupied houses and this increased to 72% in 2019. Unemployment for the eligible workforce was 4.1% in 2006 and 3.2% in 2019. In both 2006 and 2019, approximately 20% of the population resided in rural or rural/small town mix areas. 

### 2.2. Data Sources: Minnesota Pharmacies

Data were collected through biennial surveys of pharmacies located in Minnesota (Table 1) [22,23,24,25,26,27,28,29,30,31]. Each pharmacy location in Minnesota (as recorded by the Minnesota State Board of Pharmacy) was used as the unit of analysis. Outpatient pharmacies included pharmacies that were determined as being reasonably accessible by any ambulatory patient/client for receiving prescription medications and associated services. Inpatient pharmacies included hospital, institutional, and restricted-access specialty practices. In Minnesota, there were approximately 1200 pharmacies within the state. Of these, about 200 are categorized as inpatient and 1000 are categorized as outpatient.

The number of pharmacies varies from year to year due to openings, closures, mergers, and record keeping adjustments made by the State Board of Pharmacy. According to a 15-year ecological comparison of Minnesota outpatient pharmacies [33], with subsequent analysis using State Board of Pharmacy records for the year 2020, the composition of outpatient pharmacies in Minnesota changed between 2006 and 2020 and is noteworthy for this study. First, the ratio of independently owned pharmacies to chain corporation-owned pharmacies changed from 1:2 in 2007 to 1:3 in 2020. Second, the traditional retail model associated with convenient location based on population density and metropolitan designation that was utilized in 2007 changed by 2017. The ecological comparison showed that by 2017, community pharmacies shifted from the traditional retail model to health care access models based on population health needs. That is, community pharmacies were transitioning to become better organized to operate as health care access points (often associated with clinics, medical centers, or specialty centers) that provide and are reimbursed for patient care and public health services such as medication optimization, immunizations, specialized services, and more [33].

### 2.3. Data Collection

Since data were collected from key informants (owners, directors, or managers) at each pharmacy during even numbered years from 2006 through 2020, an ecological study design [34] was possible and adapted. For the years 2006 through 2018, each key informant was mailed a cover letter, a postage paid return envelope, and a questionnaire. Approximately four weeks after the initial mailing, another survey form and postage paid return envelope were mailed to non-responders. For 2020, the same process was used, but email was the delivery channel and an online response option was provided using the Qualtrics^XM^ survey platform.

In some survey years, all of the pharmacies were included in the sample and, for some years, a portion of the pharmacies were sampled for the survey. Sample sizes and response rates for each survey year are summarized below (Table 2) [22,23,24,25,26,27,28,29,30,31]:

To measure the degree of difficulty to hire pharmacists and technicians, a five point scale was used from 1 = not difficult at all to 5 = extremely difficult. Key informants were also asked to report both the ideal and realistic number of pharmacists and technicians their pharmacy would like to hire in the next year. To monitor job turnover, respondents were asked to report the number of staff members who left in the past year and the number of current open positions (for pharmacists and technicians). Finally, respondents were asked to report the hourly wage (in dollars) they would offer a newly hired pharmacist and pharmacy technician. More detailed information about the wording of these questions is available from the corresponding author (schom010@umn.edu).

As mentioned previously, five open-ended questions were added for the 2020 survey year in order to describe impactful pharmacy services, ideal characteristics of the pharmacy’s next pharmacist hire, what pharmacy staff need the most, what people served need the most, and how the COVID-19 pandemic affected their pharmacy. The Appendix A shows the wording of the open-ended questions.

### 2.4. Data Analysis

#### 2.4.1. Ecological Comparisons (2006 to 2020)

Ecological comparisons [34] were made between the survey years using descriptive statistics. Since the number of pharmacies changed from year to year, the findings reported in Table 3 and Table 4 are reported as averages or as a number per 100 pharmacies. Additionally, hourly wages are summarized both as reported and as inflation-adjusted dollars. That way, year-to-year comparisons can be more clearly interpreted by readers. For ecological studies, it should be noted that the unit of observation is the group, not separate individuals (or pharmacies).

#### 2.4.2. Open-Ended Questions in the 2020 Survey

For responses to the first question (impactful pharmacy services), categories developed by Goode and colleagues [35] were used for coding responses. For the other four open-ended questions, one of the authors (JS) developed an initial list of coding categories for content analysis of the written comments. This list was reviewed, modified, and operationalized by three researchers (S.L., A.O., J.C.S.) who met in person for this purpose. Agreement was negotiated as a valid interpretation of the text and this discussion was driven by the study objectives and consistency of emergent themes. When the final set of analyses was compared, all investigators agreed upon major themes. Triangulation, conducted by using multiple analysts, also provided a quality check on selective perception and blind interpretive bias that could occur through a single person performing all of the analysis [36]. The final categories and operational definitions for (1) impactful pharmacy services, (2) ideal characteristics of next pharmacist hire, (3) pharmacy staff member needs, (4) needs of people served, and (5) how COVID-19 affected the pharmacy are presented in the Appendix A.

For each of the other four open-ended questions, one researcher (J.C.S.) unitized the written text into a single segment for each answer from each respondent. Segments comprised the first occurrence and completeness of a concept. Three researchers (S.L., A.O., J.C.S.) were trained to conduct coding for a relatively small number of segments (*n* = 30) to assess inter-judge reliability. The researchers were trained on the rules and procedures for coding, and they independently scored each segment. Inter-judge reliabilities were then calculated by using the Perreault and Leigh reliability index (I), as follows: I = {[(F/N) − (1/k)] [k/(k − 1)]} ^1/2^, where F = the observed frequency of agreement between judges, N = the total number of judgments, and k = the number of categories [37].

The three judges had a high level of agreement (I > 0.95) except for the question “What things do the people you serve need the most right now?” which had an inter-judge reliability equal to 0.88. In light of reliability scores well above the recommended level of 0.90 for all but this question, two researchers (A.O., J.C.S.) coded this question (with S.L. serving as a tiebreaker if needed) and one researcher (J.C.S.) coded the other open-ended questions. To help assure rigor, random comments were selected and coded by three judges to make sure that consistent application of coding rules was being followed. Comparisons in the patterns of responses between outpatient and inpatient pharmacy types were made using descriptive statistics (chi-square analysis). 

## 3. Results

### 3.1. Ecological Comparisons (2006 to 2020)

Table 3 summarizes findings for outpatient pharmacies. The degree of difficulty (1 = not difficult at all to 5 = extremely difficult) to hire pharmacists has been below 3 (indicating relatively low difficulty) since 2010 in Minnesota—with the lowest score (2.1) reported in 2020. This is in contrast to the degree of difficulty to hire technicians in Minnesota being above 3 (indicating moderate difficulty) since 2014. Consistent with labor economics theory, the hourly wage for a newly hired pharmacist peaked in 2010 and then declined (using inflation adjustment to 2020 dollars). In contrast, the hourly wage for a newly hired technician increased between 2006 and 2020. Without adjusting for inflation, starting salaries for full-time pharmacists from 2006 to 2020 increased 18% and starting salaries for full-time technicians increased 44%. For reference, the Consumer Price Index (CPI) between 2006 and 2020 rose 29% [38].

Regarding hiring plans for pharmacists and the number who left in the past year, Table 3 shows year-to-year variation without strong, discernable trends over time. It is noteworthy that the proportion of full-time pharmacists increased from 69% in 2006 to 81% in 2020. Open positions for full-time pharmacists dropped from 10 per 100 pharmacies in 2006 to just 1 per 100 in 2018. Open positions for part-time pharmacists dropped from 18 per 100 pharmacies in 2006 to just 4 per 100 in 2018. The year 2020 showed an increase for part-time pharmacists (14 per 100 pharmacies), likely due to hiring for COVID-19 vaccinations.

Regarding hiring plans for technicians and the number who left in the past year, Table 3 shows year-to-year variation without strong, discernable trends over time. The composition of full-time technicians increased from 59% in 2006 to 71% in 2020. Open positions for full-time technicians increased from 12 per 100 pharmacies in 2006 to 39 per 100 in 2020. Open positions for part-time technicians increased from 27 per 100 pharmacies in 2006 to 57 per 100 in 2020. 

Table 4 summarizes findings for inpatient pharmacies. The degree of difficulty (1 = not difficult at all to 5 = extremely difficult) to hire pharmacists has been below 3 (indicating relatively low difficulty) since 2010 in Minnesota—with the lowest score (2.0) reported in 2020. This is in contrast to the degree of difficulty to hire technicians in Minnesota being at or above 3 (indicating moderate difficulty) since 2014. Consistent with labor economics theory, the hourly wage for a newly hired pharmacist peaked in 2010 and then declined (using inflation adjustment to 2020 dollars). In contrast, the hourly wage for a newly hired technician increased between 2006 and 2020. Without adjusting for inflation, starting salaries for full-time pharmacists from 2006 to 2020 increased 27% and starting salaries for full-time technicians increased 40%. For reference, the Consumer Price Index (CPI) between 2006 and 2020 rose 29% [38].

Regarding hiring plans for pharmacists and the number who left in the past year, Table 4 shows year-to-year variation without strong, discernable trends over time. The composition of full-time pharmacists increased from 68% in 2006 to 81% in 2020. Open positions for full-time pharmacists dropped from 18 per 100 pharmacies in 2006 to 9 per 100 in 2020. Open positions for part-time pharmacists dropped from 20 per 100 pharmacies in 2006 to just 2 per 100 in 2020. 

Regarding hiring plans for technicians and the number who left in the past year, Table 4 shows year-to-year variation without strong, discernable trends over time. The proportion of full-time technicians increased from 61% in 2006 to 82% in 2020. Open positions for full-time technicians increased from 10 per 100 pharmacies in 2006 to 32 per 100 in 2020. Open positions for part-time technicians increased from 12 per 100 pharmacies in 2006 to 17 per 100 in 2020. 

### 3.2. Summary of Findings for the Open-Ended Questions in the 2020 Survey

Table 5 summarizes findings from the content analysis for the five open-ended questions (refer to the Appendix A for categories and operational definitions). For outpatient pharmacies, the most impactful services were Medication Optimization and Wellness/Prevention services. For inpatient pharmacies, the most impactful services were Medication Optimization and Public Health. 

The distribution of “ideal characteristics of your next pharmacist hire” was statistically different for outpatient and inpatient pharmacies. For outpatient (community-based) pharmacies, the ideal characteristics were Specialized Experience/Training (43%) followed by Work Ethic (35%) and Interpersonal Skills (21%). For inpatient pharmacies, the percentages (proportions) were significantly different: Specialized Experience/Training (75%) followed by Work Ethic (22%) and Interpersonal Skills (3%).

Two (2) representative written comments for this question included:

*Can effectively communicate, embraces treatment modalities such as: Rx, OTC, dietary supplements, dietary components to health, chiropractic and kinesio therapies, acupuncture, and other non-drug therapies. Hunger for learning. Innovative in thought and process. Collaborative in nature.*

*Residency trained, hospital experience, willingness to work weekends, evenings, holidays. Comfortable with face to face patient interactions. Additional certifications preferred (BCPS, ACLS, PALS; ASHP certificates).*

Regarding pharmacy staff member needs, both types of pharmacies (inpatient and outpatient) reported the same things: Staff Support (64%), Staff Training (29%), and Health System Reforms (7%). Two (2) representative written comments for this question included:

*Budgets are tight. We need to ensure items are properly billed to avoid audits and are utilizing the systems in place to ensure insurance companies are giving us preferred status. Additional training could be helpful. Additional staff would be helpful, but not currently possible due to budget constraints.*

*More help/support from corporate. The expectations are hard to accomplish with the limited resources provided from corporate with the most important resource being more budget hours for staffing. More management training. Most new pharmacists have minimal training and therefore desire to manage people.*

Regarding what the people they serve need right now, both types of pharmacies reported the same things: Access to Care (43%), Quality Care (27%), Affordable Care (21%), and Social Justice (9%). Two (2) representative written comments for this question included:

*A pharmacy that they can come to and not worry about the cost of their medications—if they are preferred or not. They need someone who listens to them and can help them with their needs and how to navigate the health care system—they need an advocate in the health system. Home delivery so that they can get their medications during this pandemic.*

*Healthcare reform. Medicare is a mess with preferred contracts and DIR being unsustainable. Costs are running out of control forcing increased price sensitivity and patients falling in to polypharmacy traps that decrease safety. Pharmacies need to be incentivized to provide high quality services that improve patient care; NOT filling as many Rx’s as possible for the lowest price possible. PBM’s need to provide value and the current model does not; too many dollars are leeched out of healthcare and while not the only problem, they are a big one.*

We would be remiss if we did not provide examples of comments related to Social Justice. Examples for this category included:

*Providers/systems that care enough to take the time necessary to achieve positive/life changing outcomes. Someone who will address all aspects of health from a mind, body, spirit perspective. Dynamic providers that can fill in deficiencies that are unique to each patient and situation. Suitable living arrangements. Education. Jobs. Financial assistance. Safe environment. Trustworthiness. Understanding. A focus on population health strategies- focusing on community change-adding more walking trails, community gardens, exercise facilities, youth facilities. Activities to keep kids from going the route of drug addiction (more youth focused activities & outlets).*

When asked about how the COVID-19 pandemic affected their pharmacy, most responses related to Service Access and Coordination (33%), followed by Staff Workloads and Stress (26%), Revenues and Expenses (23%), and Regulations and Precautions (17%). Two (2) representative written comments for this question included:

*Changing to a curbside/delivery/shipping model to keep staff and business safe has increased the workload for all of us. Expenses are up at the same time reimbursements are at an all time low. Not enough resources to continue to safely and effectively carry on much longer. Trying to figure out our next move to offset additional reimbursement cuts coming with the 2021 PBM contracts. We are all exhausted trying to keep up with the ever increasing workload caused by pharmacy closings due to economic and civil unrest issues.*

*Leveraging more remote capabilities than planned at this point, escalated our strategic plan. Has us reimagining physical structure needs of the future. The pandemic will no doubt speed up tools and investigations already ongoing into more autonomous and augmented workflows. Virtual care team structures and the tools/applications that go along with supporting that continue to advance.*

We limited the number of examples for each open-ended question but received many insightful comments. For readers who are interested in seeing more written comments to the open-ended questions, please contact the corresponding author at schom010@umn.edu.

## 4. Discussion

Before the findings are discussed, study limitations should be acknowledged. First, workforce data were collected from licensed Minnesota pharmacies and do not include other work settings in which pharmacists are employed. Since we only collected data from licensed pharmacies, our findings do not represent job characteristics in other settings. Second, we received responses from only a portion of the pharmacies in Minnesota. Our estimates are based on the assumption that respondents to our survey were representative of Minnesota pharmacies. Third, the number and mix of various types of pharmacies coded as inpatient or as outpatient could have changed from year to year and could affect comparisons over time. Fourth, ecological comparisons were made with group level information. To avoid errors of ecological fallacy, inferences should not be made about individual pharmacies from the results of aggregate data [34]. Finally, Minnesota has unique characteristics such as: (1) relatively highly integrated health care systems that include pharmacists in collaborative care, (2) community pharmacy practice transformation into centers for health and personal care, (3) specialty services development in pharmacies, and (4) challenging economic pressures placed upon pharmacies by other organizations in the health care value chain. Not all of these characteristics may apply to other regions to the same extent.

Between 2006 and 2020, the findings showed that the market for jobs in licensed pharmacies in Minnesota transitioned from a slight undersupply to an oversupply of pharmacists. During that same period, the technician labor market transitioned from a slight oversupply to a moderate undersupply of technicians. Trends in salaries are consistent with labor economic principles that showed pharmacist salaries grew at a lower rate and technician salaries grew at a higher rate than the Consumer Price Index (CPI).

The higher demand for technicians might be due to their expanding roles that have enabled them to conduct work activities traditionally reserved for the pharmacist, but at less cost, in order to meet economic challenges in current pharmacy practice [39]. Furthermore, responses to our open-ended questions suggest that pharmacies are transforming their work systems and processes of care for the next generation of pharmacy practice [40] and (1) seek pharmacists with specialized experience and training, (2) acknowledge the need for expanded staff support, and (3) are focusing on creating better patient access to their service offerings. Thus, in addition to typical frictional forces (job turnover), the hiring dynamics of Minnesota pharmacists have been affected by structural unemployment forces that require new skills for pharmacists and transitions to technologies and technicians for fulfilling traditional pharmacist work activities [13,14,15].

While the findings show that Minnesota pharmacies would like to hire more pharmacists into roles that require new skills, survey respondents articulated that this is not feasible under current economic pressures. In addition, respondents described how economic pressures not only limit expansion of pharmacist roles but also are creating understaffing and job stress. Thus, an oversupply of pharmacists may exist due to economic pressure and organizational responses to that pressure rather than a lack of demand for the expanded services that pharmacists can provide. This may represent a lost opportunity, resulting in understaffing at pharmacies that not only adversely affects pharmacy staff wellbeing/resilience but also patient safety [41].

We expect this labor market to remain dynamic in light of uncertainty surrounding: (1) the U.S. political economy, (2) effects of health care reform, (3) social justice and health equity transformation, (4) new job opportunities in organizations that are not licensed as pharmacies, (5) the use of technicians as less expensive substitutes for some pharmacist work activities, and (6) abrupt adjustments in business models and practices for existing pharmacies. In addition to typical functional unemployment forces (job turnover), we expect structural unemployment to also be a force that drives the need for new skills training.

## 5. Conclusions

The findings showed an increased demand for technicians in Minnesota pharmacies which might be due to their expanding roles that have enabled them to conduct work activities traditionally reserved for the pharmacist, but at less cost, in order to meet economic challenges in current pharmacy practice [39]. Minnesota pharmacies reportedly would like to hire pharmacists to meet the demand for new services that pharmacists can provide. However, respondents articulated that this is not feasible under current economic pressures. This represents a lost opportunity for transformation in pharmacy that would establish pharmacists’ roles in the rapidly transforming health care value chain. Respondents also described how economic pressures are resulting in understaffing at pharmacies that adversely affects pharmacy staff wellbeing/resilience and patient safety. We conclude that hiring dynamics in pharmacies are being driven more by economic and organizational shifts than meeting the demand for services that pharmacists can provide.

## Figures and Tables

**Table 1:** Sample Sizes for Biennial Surveys of Minnesota Pharmacies.

Year	Number Inpatient	Number Outpatient	Total
2006	171	1042	1213
2008	179	1060	1239
2010	178	1035	1213
2012	156	1077	1233
2014	214	1043	1257
2016	174	1089	1263
2018	209	1053	1262
2020	191	930	1121

**Table 2:** Response Rates for Biennial Surveys of Minnesota Pharmacies.

Year	Overall Response Rate	Number of Usable Surveys	Number Inpatient	Number Outpatient
2006	733/1213 = 60%	716	100	616
2008	783/1239 = 63%	766	124	642
2010	673/1213 = 55%	657	130	527
2012	613/1233 = 50%	613	113	500
2014	438/1257 = 35%	434	74	360
2016	242/530 = 46%	242	98	144
2018	237/544 = 44%	237	97	140
2020	322/670 = 48%	322	138	184

**Table 3:** Pharmacist and Technician Hiring Characteristics for Outpatient ^a^ Minnesota Pharmacies (*n* = 3113).

For standardization of results, findings are reported as “number per 100 pharmacies” or as averages								
	**2006**	**2008**	**2010**	**2012**	**2014**	**2016**	**2018**	**2020**
Degree of difficulty ^b^ to hire pharmacists (both full time and part time)	3.7	3.3	2.5	2.5	2.6	2.5	2.3	2.1
**Pharmacists: Full Time (at least 30 h per week)**								
Number Employed	204	219	217	202	225	194	229	318
Ideal Number to be Hired in the Next Year	24	18	8	12	12	7	8	28
Realistic Number Hired in the Next Year	9	11	4	4	5	5	4	9
Number Who Left in the Past Fiscal Year	30	27	23	24	34	24	19	52
Number of Current Open Positions	10	12	2	2	4	6	1	2
Hourly Wage for Newly Hired Person	$48.48	$52.30	$54.39	$55.84	$56.43	$57.65	$60.03	$57.38
Inflation-Adjusted ^c^ to 2020 Dollars	$62.24	$62.87	$64.56	$62.95	$61.69	$62.17	$61.87	$57.38
**Pharmacists: Part Time (<30 h per week)**								
Number Employed	93	92	91	90	107	83	91	76
Ideal Number to be Hired in the Next Year	46	37	24	39	27	31	26	45
Realistic Number Hired in the Next Year	19	15	9	10	19	6	8	11
Number Who Left in the Past Fiscal Year	17	15	13	15	13	12	19	32
Number of Current Open Positions	18	14	4	7	7	6	4	14
Hourly Wage for Newly Hired Person	$48.27	$51.86	$53.68	$55.60	$56.74	$57.46	$59.76	$56.60
Inflation-Adjusted ^c^ to 2020 Dollars	$61.97	$62.34	$63.71	$62.68	$62.03	$61.96	$61.69	$56.60
Degree of difficulty ^b^ to hire technicians (both full time and part time)	3.0	2.8	2.6	2.8	3.2	3.5	3.6	3.3
**Technicians: Full Time (at least 30 h per week)**								
Number Employed	267	308	270	275	330	291	327	441
Ideal Number to be Hired in the Next Year	42	48	19	24	29	37	34	84
Realistic Number Hired in the Next Year	18	20	8	11	20	21	21	44
Number Who Left in the Past Fiscal Year	46	49	34	42	61	66	78	90
Number of Current Open Positions	12	12	9	7	13	18	27	39
Hourly Wage for Newly Hired Person	$10.77	$10.89	$11.72	$11.83	$12.88	$13.53	$14.58	$15.54
Inflation-Adjusted ^c^ to 2020 Dollars	$13.83	$13.09	$13.91	$13.34	$14.08	$14.59	$15.03	$15.54
**Technicians: Part Time (<30 h per week)**								
Number Employed	189	156	181	202	185	159	194	177
Number Who Left in the Past Fiscal Year	60	57	35	42	59	58	70	63
Ideal Number to be Hired in the Next Year	65	48	52	59	58	58	63	73
Realistic Number Hired in the Next Year	51	48	52	59	58	58	63	73
Number of Current Open Positions	27	23	14	19	27	33	39	57
Hourly Wage for Newly Hired Person	$10.34	$10.44	$11.28	$11.60	$12.54	$13.33	$14.32	$15.25
Inflation-Adjusted ^c^ to 2020 Dollars	$13.27	$12.55	$13.39	$13.08	$13.71	$14.37	$14.76	$15.25

Data source: Minnesota Biennial Pharmacy Surveys (2006, 2008, 2010, 2012, 2014, 2016, 2018, 2020). ^a^ Outpatient pharmacies included pharmacies that were determined as being reasonably accessible by any ambulatory patient/client for receiving prescription medications and associated services. ^b^ Degree of difficulty (1 = not difficult at all to 5 = extremely difficult). ^c^ Inflation-adjusted calculations computed at: https://www.usinflationcalculator.com/, accessed 17 February 2021.

**Table 4:** Pharmacist and Technician Hiring Characteristics for Inpatient ^a^ Minnesota Pharmacies (*n* = 874).

For standardization of results, findings are reported as “number per 100 pharmacies” or as averages								
	**2006**	**2008**	**2010**	**2012**	**2014**	**2016**	**2018**	**2020**
Degree of difficulty ^b^ to hire pharmacists (both full time and part time)	3.7	3.5	2.8	2.6	2.6	2.9	2.6	2.0
**Pharmacists: Full Time (at least 30 h per week)**								
Number Employed	559	682	668	766	799	497	501	883
Ideal Number to be Hired in the Next Year	53	65	35	50	53	42	29	50
Realistic Number Hired in the Next Year	35	36	19	31	35	21	15	21
Number Who Left in the Past Fiscal Year	37	46	40	59	77	29	32	40
Number of Current Open Positions	18	32	12	76	36	13	7	9
Hourly Wage for Newly Hired Person	$46.02	$49.74	$51.62	$53.72	$54.56	$56.42	$57.90	$58.51
Inflation-Adjusted ^c^ to 2020 Dollars	$59.08	$59.79	$61.27	$60.56	$59.65	$60.84	$59.68	$58.51
**Pharmacists: Part Time (<30 h per week)**								
Number Employed	258	209	226	229	196	168	165	203
Ideal Number to be Hired in the Next Year	54	56	38	34	42	30	29	25
Realistic Number Hired in the Next Year	25	22	19	15	15	17	14	9
Number Who Left in the Past Fiscal Year	15	14	18	14	26	12	12	18
Number of Current Open Positions	20	22	7	6	4	6	7	2
Hourly Wage for Newly Hired Person	$46.90	$49.61	$51.66	$53.91	$54.65	$56.34	$58.21	$58.67
Inflation-Adjusted ^c^ to 2020 Dollars	$60.21	$59.64	$61.32	$60.77	$59.75	$60.75	$60.00	$58.67
Degree of difficulty ^b^ to hire technicians (both full time and part time)	2.6	2.6	2.4	2.8	3.1	3.5	3.5	3.0
**Technicians: Full Time (at least 30 h per week)**								
Number Employed	533	714	702	744	847	518	478	753
Ideal Number to be Hired in the Next Year	54	56	30	47	65	44	49	84
Realistic Number Hired in the Next Year	30	45	24	27	43	22	41	53
Number Who Left in the Past Fiscal Year	49	70	70	63	55	83	67	87
Number of Current Open Positions	10	14	13	20	15	17	27	32
Hourly Wage for Newly Hired Person	$12.71	$13.81	$13.88	$14.53	$14.41	$15.59	$17.10	$17.83
Inflation-Adjusted ^c^ to 2020 Dollars	$16.32	$16.60	$16.47	$16.38	$15.75	$16.81	$17.62	$17.83
**Technicians: Part Time (<30 h per week)**								
Number Employed	337	297	310	320	259	200	155	167
Ideal Number to be Hired in the Next Year	60	45	44	43	38	46	45	38
Realistic Number Hired in the Next Year	37	34	28	25	21	26	26	26
Number Who Left in the Past Fiscal Year	45	54	48	44	12	41	42	47
Number of Current Open Positions	12	12	15	17	6	13	14	17
Hourly Wage for Newly Hired Person	$12.53	$13.56	$13.42	$14.35	$14.36	$15.57	$16.45	$17.69
Inflation-Adjusted ^c^ to 2020 Dollars	$16.09	$16.30	$15.93	$16.18	$15.70	$16.79	$16.95	$17.69

Data source: Minnesota Biennial Pharmacy Surveys (2006, 2008, 2010, 2012, 2014, 2016, 2018, 2020). ^a^ Inpatient pharmacies included hospital, institutional, and restricted-access specialty practices. ^b^ Degree of difficulty (1 = not difficult at all to 5 = extremely difficult). ^c^ Inflation-adjusted calculations computed at: https://www.usinflationcalculator.com/, accessed 17 February 2021.

**Table 5:** Content Analysis Summary for Open-Ended Questions Contained in the 2020 Survey (*n* = 322 since these questions only were included for the 2020 survey).

	Outpatient Pharmacies	Inpatient Pharmacies
What are the most impactful services available at your pharmacy? (sums total more than 100% due to multiple responses)	Medication Optimization (88%) Wellness and Prevention (72%) Public Health (28%) Patient Support Services (27%) Patient Education (23%) Chronic Care Management (20%) Acute Care Management (14%) Monitoring/Laboratory Testing (11%)	Medication Optimization (79%) Public Health (40%) Patient Education (35%) Monitoring/Laboratory Testing (27%) Patient Support (25%) Chronic Care Management (25%) Wellness and Prevention (19%) Acute Care Management (13%)
What are the ideal characteristics of your next pharmacist hire? (Chi-square, *p* < 0.001)	Specialized Training/Experience (43%) Work Ethic (36%) Interpersonal Skills (21%)	Specialized Training/Experience (75%) Work Ethic (22%) Interpersonal Skills (3%)
What things do your pharmacy staff members need the most right now? (Chi-square, *p* = 0.07)	Staff Support (66%) Staff Training (25%) Health System Reforms (9%)	Staff Support (62%) Staff Training (36%) Health System Reforms (2%)
What things do the people you serve need the most right now? (Chi-square, *p* = 0.16)	Access to Care (48%) Quality Care (24%) Affordable Care (21%) Social Justice (7%)	Access to Care (33%) Quality Care (33%) Affordable Care (20%) Social Justice (14%)
How has the COVID-19 pandemic affected your pharmacy? (Chi-square, *p* = 0.15)	Service Access/Coordination (37%) Revenues/Expenses (23%) Staff Workload/Stress (21%) Regulations/Precautions (19%)	Staff Workload/Stress (35%) Service Access/Coordination (27%) Revenues/Expenses (24%) Regulations/Precautions (14%)

Refer to the Appendix A for a description of the categories used for content analysis.

## Data Availability

Data files are stored in encrypted format at the University of Minnesota. Requests for access to the files may be sent to the corresponding author at schom010@umn.edu.

## Appendix A. Categories and Operational Definitions for Content Analysis

What are the most impactful services available at your pharmacy [35]?

Medication Optimization: Medication Packing, Home Delivery, Medication Reconciliation, Appointment-Based Medication Reconciliation, Medication Adherence Programs, Comprehensive Medication Management Services, Targeted Medication Review, Medication Administration, De-prescribing, Medication Synchronization/Auto Refill Programs, Compounding.

Wellness and Prevention: Screenings, Risk Assessment, Weight Management, Tobacco Cessation, Contraception, Bioidentical Hormone Replacement, Fluoride Treatments, Naloxone, Needle Exchange, Drug Take Back, Nutraceuticals, Annual Wellness, Pharmacogenomics, Sleep Assessment, Falls Prevention, Immunization, Pre-Travel Health Services.

Chronic Care Management: Diabetes, Hypertension, Cholesterol, Asthma, Anticoagulation, Heart Failure, Hepatitis C, Menopause, Pain Management.

Monitoring through Laboratory Testing: Bioidentical hormone replacement therapy (BHRT) saliva testing, Anticoagulation, A1c, TSH (thyroid stimulating hormone), Free thyroxine, Pharmacogenetics, Liver function.

Acute Care Management: Examples include: Test and Treat (rapid diagnostics for Influenza, COVID-19, Strep, H. pylori), Urgent Care (minor ailments), Triage and Referral.

Patient Education: Education resources located in the pharmacy, Patient Language/Communication Services, Individual Education, Group Education, Exercise Classes/Yoga.

Patient Support Services: Telehealth, Stay-in-Home Patient Care Services, Durable Medical Equipment, Care Transitions, Drive Thru/Curb Side Pickup Services, Medical Equipment, Oxygen/Continuous Positive Airway Pressure (CPAP) services, Home Care, Infusion Services.

Public Health: Population Health, Emergency Preparedness, Practice-Based Research, 340B Drug Pricing Program, Collaborative Practice, Team-based/Collaborative Care.

What are the ideal characteristics of your next pharmacist hire?

Specialized Training/Experience: Training, experience, and abilities that are specifically suited to the needs of the organization.

Work Ethic: Ability and motivation to be flexible, efficient, and focused to meet the changing needs of the organization.

Interpersonal Skills: Ability and motivation to be aware (of self and others), caring, and collaborative. Strong communication and conflict management skills.

What things do your pharmacy staff members need the most right now?

Staff Support: Staffing, workload, technology, quality of work life.

Staff Training: Opportunities for learning new skills and for applying abilities.

Health System Reforms: Changes to burdensome insurance, benefit management, regulatory, and reimbursement schemes.

What things do the people you serve need the most right now?

Access to Care: Locational convenience and communication connectivity for obtaining medications, advice, and services.

Quality Care: Tailored, accurate, responsive, reliable, stable, and consistent care with emotional support and continuity.

Affordable Care: Financial assistance, affordability, and insurance payment reform.

Social Justice: Education, housing, safety, jobs.

How has the COVID-19 pandemic affected your pharmacy?

Service Access/Coordination: Work System and Care Process changes to provide access and coordination.

Revenues/Expenses: Both increases and decreases in revenues and expenses.

Staff Workload/Stress: Increases in staff workload and stress.

Regulations/Precautions: Addressing new regulations and implementing safety precautions.
